# A Novel Differential High-Frequency Current Transformer Sensor for Series Arc Fault Detection

**DOI:** 10.3390/s19173649

**Published:** 2019-08-22

**Authors:** Guanghai Bao, Xiaoqing Gao, Run Jiang, Kai Huang

**Affiliations:** 1 College of Electrical Engineering, Fuzhou University, Fuzhou 350108, China; 2 Fujian Key Laboratory of New Energy Generation and Power Conversion, Fuzhou 350108, China

**Keywords:** arc, arc fault detection, differential high-frequency current transformer (D-HFCT), high-frequency noise, ferrite core, differential threading method

## Abstract

Fault arc detection is an important technology to ensure the safe operation of electrical equipment and prevent electrical fires. The high-frequency noise of the arc current is one of the typical arc characteristics of almost all loads. In order to accurately detect arc faults in a low-voltage alternating-current (AC) system, a novel differential high-frequency current transformer (D-HFCT) sensor for collecting high-frequency arc currents was proposed. The sensitivity and frequency band of the designed sensor were verified to ensure that the acquisition requirements of the high-frequency current were satisfied. A series arc fault simulation experiment system was built, and resistive, inductive, and non-linear load and high-power shielding load experiments were carried out. Experiments showed that the sensor output signal was close to zero in the non-arc state, and the sensor output response was a high-frequency glitch in the arc state. The results were consistent for different loads, and the discrimination between normal and fault states was obvious, which proved that the sensor is suitable for series arc fault detection.

## 1. Introduction

With the rapid development of China’s electric power industry, various types of electrical equipment have become indispensable in people’s living and production practices, but the problem of electricidal safety cannot be ignored. According to statistics from the Fire Department of the Ministry of Public Security, 237,000 fires occurred in 2018, resulting in a total loss of 3.675 billion yuan. In terms of the causes of the fires, 82,000 fires were caused by electricity, accounting for 34.6% of the total. Electrical fires are the main cause of fires [1]. In general, short-circuit fault, overload fault, earth leakage fault, and arc fault are the primary causes of electrical fire emergencies. The first three types of faults can be detected and protected against by circuit breakers, and fuse and leakage protectors, respectively [2,3,4]. However, these devices often cannot detect arc faults completely.

According to the Standard IEC 62606-2017, an arc is defined as the phenomenon of luminous discharge across an insulating medium, which is usually accompanied by partial volatilization of the electrodes. An arc fault is defined as a dangerous unintentional arc [5]. Generally, arc faults can be classified into three types: earth arc fault, parallel arc fault, and series arc fault [5,6]. Since the current increases rapidly when the first two types of faults occur, the protection devices can easily detect these faults and remove the fault section. Due to the limitation of load impedance, when a series arc fault occurs, the arc current is not much different from the normal operating current, which means that conventional power protection devices cannot provide protection [2,3,4]. Studies have shown that the temperature of a series fault arc can reach 5000 to 15,000 °C. Aging electrical equipment wiring, damaged electrical insulation, and poor contact can cause arcing faults that cause electrical fires due to the release of large amounts of heat. Therefore, effective and reliable series arc fault detection of power systems is of great significance to prevent the occurrence of electrical fires and protect people’s lives and property.

In early studies, some scholars put forward improved arc models and simplified arc models [7,8,9]. However, since the arc process is a multi-physics coupling process, the situation is extremely complex. Therefore, the arc mathematical model is suitable for theoretical analysis, but it is not practical in arc fault detection.

An arc is a gas discharge phenomenon accompanied by changes in sound, light, heat, electromagnetic fields, and temperature. In Reference [10], a pressure zone microphone, an infrared receiver, and a loop antenna were used to detect changes in pressure, temperature, and electromagnetic fields, respectively. In Reference [11], a stick antenna and loop antenna were used to detect electromagnetic radiation signals generated by arc faults. References [12,13] used both electric field sensors and magnetic field sensors to capture abnormal electric and magnetic signals generated by arc faults. These methods are suitable in cases where the arc fault location is determined. However, due to the randomness of the arc location, these methods are not applicable in practice.

At present, the development of machine learning (ML) and artificial intelligence (AI) has made many excellent artificial intelligence algorithms the focus of people’s research, such as artificial neural networks (ANNs) and support vector machines (SVMs). Reference [14] proposed a comprehensive approach of complex load recognition and series arc detection based on a principle component analysis and support vector machine (PCA-SVM) combination model. Reference [15] developed deep neural networks (DNNs) taking Fourier coefficients, Mel-frequency cepstrum data, and Wavelet features as input for differentiating normal from malignant current measurements. Reference [16] used the approach of a radial basis function neural network (DRBFNN) to identify the occurrence of series arc faults. However, these intelligent algorithms are complex and require a large amount of computer software and hardware resources, so it is difficult to implement actual product applications at present, and they are mostly in the stage of theoretical analysis and research.

The research methods for current time–frequency, frequency–domain, and time–frequency domain signals are still the focus of AC arc fault detection methods. In Reference [17], a high-resolution low-frequency harmonic analysis method based on chirp zeta transform (CZT) and a series of indicators were proposed to detect arc faults. Reference [18] proposed a multi-index arc detection method by summarizing the volt-ampere characteristics of arc under different loads. Reference [19] designed a band-pass filter with a frequency of 2.4 to 39 kHz to extract the arc signal based on the elimination of low-frequency power signals and high-frequency load noise.

**Figure 1:**
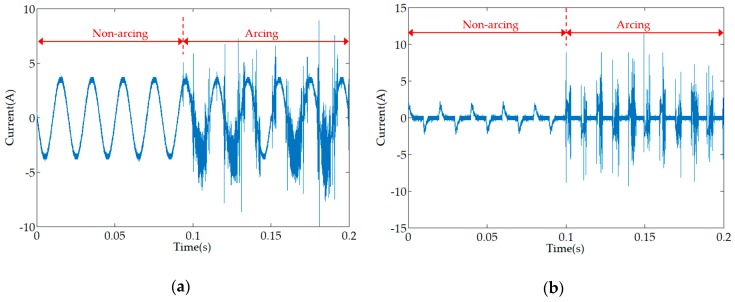
The current waveforms of several loads: (**a**) incandescent lamp; (**b**) energy saving lamp.

The current waveforms of two common low-voltage appliances in non-arc and arc states are shown in Figure 1. When an arc fault occurs in the circuit, the current waveform in the line will be significantly distorted [20]. The detection and analysis of the current signals can effectively identify the AC series arc faults. Many tests have shown that the typical characteristics of arc faults are the flat shoulder and high-frequency noise of the current waveform [21,22,23]. For resistive loads, their flat shoulders are more obvious, and it is easy to distinguish the fault signal. However, for inductive and non-linear loads, such as an air compressor, halogen lamp, vacuum cleaner, and microwave oven, their waveform distortion is severe, and it is easy to cause misjudgment and missed judgment from the characteristics of the flat shoulder [24]. In addition, if the high-power branch is connected in parallel with the low-power fault branch, the fault signal is easily submerged, and it is difficult to determine the arc fault [20]. High-frequency noise, which is one of the typical features of arc faults, is often considered to be one of the effective arc fault detection methods [21,23,24]. In previous papers and tests, the arc fault current is rich in high-frequency noise, and its frequency can reach hundreds of kHz or even tens of MHz [24,25]. Therefore, the high-frequency noise of the arc current can be used as the basis for the occurrence of arc faults. The key to arc fault detection lies in the acquisition of high-frequency current signals.

A current transformer (CT) with a silicon steel core can collect low-frequency signals. The low-frequency signal of the current reflects the overall trend of the current and there are many signal waveforms in the power systems that approximate arc faults. Due to the wide variety of electrical appliances and new innovations, it is difficult to find the universally applicable feature quantities (such as slope and variance) of low-frequency signals, which makes it difficult to distinguish the normal state and arc fault state of the circuit from the low-frequency waveform. Currently, air core coils (Rogowski coils) are usually used to measure broadband, transient currents. [26,27]. In order to increase the magnitude of the output response and make the sensor operating frequency in the high-frequency band, some scholars have proposed using high-frequency magnetic materials such as the core of the air-core coil. These types of sensors are called high-frequency current transformer (HFCT) sensors. In the high-frequency band, the magnetic permeability of the core material of the HFCT sensor is several hundred times or even several thousand times larger than the vacuum magnetic permeability, which can improve the output response and effectively collect high-frequency signals. However, the high-frequency core has a non-linear change in magnetic permeability under alternating high current conditions, which limits the application of the HFCT sensor. [28,29,30,31]. If the core is saturated, the magnetic permeability will decrease rapidly, close to the permeability of the air. The smaller the measured current or the magnetic field strength of the core material, the closer the magnetic permeability is to the initial permeability (the initial permeability is a constant) [30,31]. That is to say, under low-magnetic flux density, the non-linearity of the high-frequency magnetic material is low, and the magnetic permeability is relatively constant.

Due to the limitation of the load impedance, the current value of the series arc fault ranges from 5 Amperes to 30 Amperes [32]. For HFCT sensors, large currents produce large fluxes that cause the core to operate away from the linear working area. As the current changes, the magnetic permeability of the core changes non-linearly, causing severe distortion of the output waveform. Therefore, few papers have proposed the use of HFCT for arc fault detection. A common measure is to process the acquired analog signal through a complex algorithm (wavelet decomposition algorithm) or a filter circuit.

Suppose there are two parallel branches, one is a low-power arc fault branch and the other is a high-power normal branch. The current flowing through the main road is the sum of the currents of the two parallel branches. However, it is difficult to detect an arc fault on the main road because the current of the small power arc fault branch is negligible compared to the current of the high-power normal branch. We call this phenomenon the shielding effect of the high-power branch. The sensor placed on the main line cannot detect the occurrence of an arc fault and causes a missed judgment. However, it is uneconomical to place sensors on all branches to detect arc faults, and we expect sensors placed on the main line to detect all branch arc faults within its protection range.

In this paper, a novel high-frequency current sensor based on the differential threading method was put forward and used in low-voltage series arc fault detection. In Reference [33], the residual magnetic flux caused by the asymmetry of the position of the live line and the neutral line was proposed as the measured physical quantity. However, the limitation of this method is that the asymmetry of the live and neutral lines is based on the fact that the secondary windings are not evenly wound around the entire core. Although that any winding method is not perfect is a well-known fact. In Reference [33], no detailed analysis and explanation of the structure and material of the current transformer was made, but the key to this method lays in the structure of the transformer and the core material. In this paper, a non-uniform current sensor with differential threading method is proposed, which is a further improvement of the sensor used in Reference [33]. Through numerical analysis, the influence of the structure of the sensor and the core material on the transmission characteristics of the sensor was quantitatively studied. The actual arc detection effect of the sensor was tested by self-made sensors and different single-load experiments and high-power shielding load experiments on the established arc experiment platform. 

The differential threading method of HFCT proposed in this paper has two advantages:

(1) The amplitude of the equivalent magnetic flux induced by the high-frequency magnetic core is reduced, so that the core material works in the linear working area, and the waveform is hardly distorted. In this way, the sensor can acquire the high-frequency arc fault signal.
(2) For the fault signal shielding problem of the low-power fault branch caused by the high-power branch, the sensor placed on the main line can extract the low-power arc current signal because of the attenuation effect of the high-frequency core material on the low-frequency signal and the offset effect of the differential threading method on the magnetic flux.

This article is divided into five sections. Section 2 illustrates the structural characteristics and working principle of the D-HFCT sensor; Section 3 presents the equivalent circuit and transmission characteristics of the D-HFCT sensor and numerically analyzes the influence of the eccentricity of the primary conductor and the secondary windings parameters on the sensitivity of the sensor by means of commercial data software (matrix laboratory); Section 4 verifies the D-HFCT sensor and designs a series arc fault simulation experiment system and various load experiments were carried out to test the practicability of the D-HFCT sensor. Finally, the conclusions and prospects of this paper are given in Section 5.

## 2. Structure and Characteristics of the D-HFCT Sensor

### 2.1. Structure

**Figure 2:**
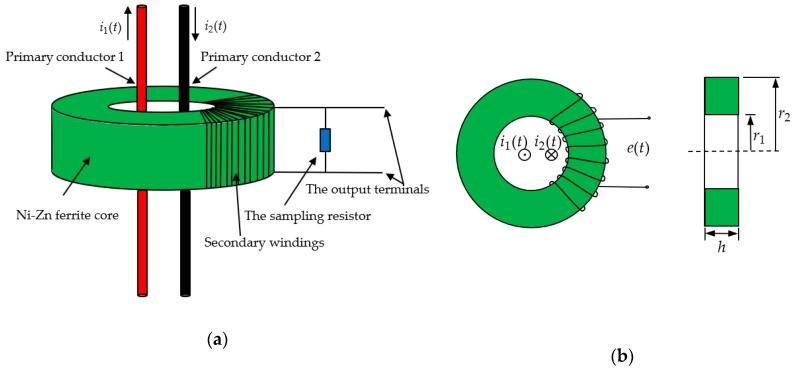
(**a**) Schematic diagram of a differential high-frequency current transformer (D-HFCT) sensor; (**b**) dimensions of a D-HFCT sensor (without a sampling resistor).

The schematic of the D-HFCT sensor is shown in Figure 2a. Unlike the current transformer (CT), the angle of the secondary windings wrapped around the core was less than 360°, and the primary conductors passing through the current sensor were differentially threaded. The currents $i_{1}(t)$ and $i_{2}(t)$ flowing through the primary conductors were equal in magnitude and opposite in direction. Figure 2b shows the dimensions of a D-HFCT sensor. e(t) is the induced voltage on the secondary windings of the sensor. The circular core of the sensor adopted a rectangular cross-section, the height of the rectangular section is *h*, the inner radius is $r_{1}$, the outer radius is $r_{2}$, and the number of turns of the coil is N.

**Figure 3:**
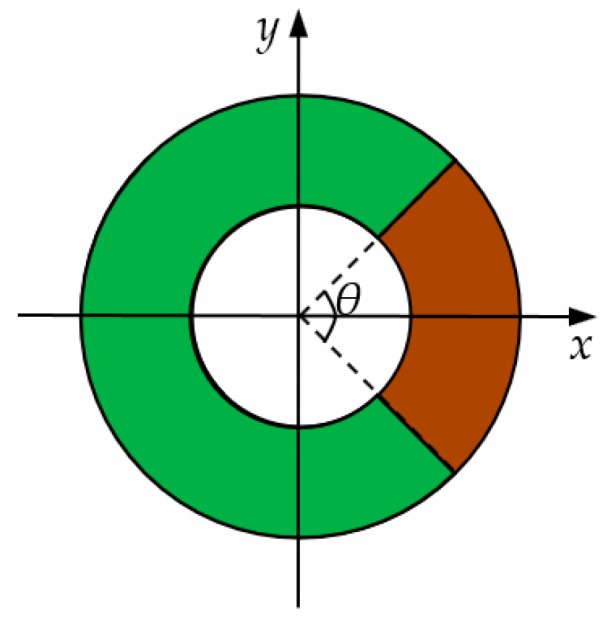
Schematic diagram of the secondary windings.

The schematic diagram of the secondary windings is shown in Figure 3. The secondary windings used a centralized distribution pattern, which is different from the common uniform distribution pattern, and the windings were concentrated at a shaded area of angle θ, where $0^{{}^{\circ}}<\theta <360^{{}^{\circ}}$. To simplify the analysis, this paper assumed that the secondary windings were symmetrically distributed in (−θ/2, θ/2) and two primary conductors were placed perpendicular to the plane of the coil along the *x*-axis and placed on either side of the *y*-axis.

**Figure 4:**
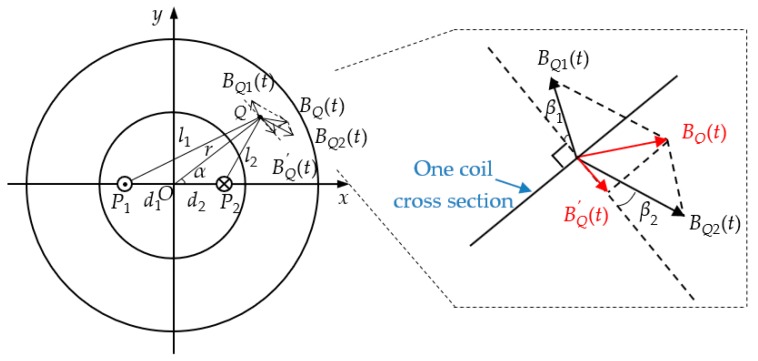
Magnetic field generated by primary conductors.

The magnetic field generated by the primary conductors is shown in Figure 4. Point *Q* is any point on the core and r is the distance from point *Q* to the center of the circle. The primary conductor 1 and the primary conductor 2 were placed at point $P_{1}$ and point $P_{2}$, respectively. $d_{1}$ and $d_{2}$ are the distances from point $P_{1}$ and point $P_{2}$ to the center of the circle, and $l_{1}$ and $l_{2}$ are the distances from point $P_{1}$ and point $P_{2}$ to point *Q*, $∠QOP_{2}=\alpha$.

According to the Biot–Savart Law, the magnetic flux density produced by primary conductors at point *Q* can be expressed as follows:

(1) $$B_{Q1}(t)=\frac{\mu i_{1}(t)}{2\pi l_{1}}$$

(2) $$B_{Q2}(t)=\frac{\mu i_{2}(t)}{2\pi l_{2}}$$

where μ is the permeability of the ferrite core, $\mu =\mu_{0}\mu_{r}$ and $\mu_{0}$ are the permeability of vacuum, and $\mu_{r}$ is the relative permeability of the ferrite material.

According to the resultant vector algorithm, the resultant flux density at point *Q* can be:

(3) $$\vec{B_{Q}(t)}=\vec{B_{Q1}(t)}+\vec{B_{Q2}(t)}$$

The vertical component of the resultant flux density on the secondary windings section is:

(4) $$B_{Q}^{\prime }(t)=B_{Q2}(t)\cos\beta_{2}-B_{Q1}(t)\cos\beta_{1}$$

where $\beta_{1}$ is the angle between $B_{Q1}(t)$ and the vertical direction of the section, $\beta_{2}$ is the angle between $B_{Q2}(t)$ and the vertical direction of the section. The purpose of introducing the vertical component of the resultant flux density was to derive the magnetic flux through the cross-section of the coil.

According to the geometric relationship and the cosine theorem, $∠OQP_{1}=\beta_{1}$, and $∠OQP_{2}=\beta_{2}$, the following equations can be derived:

(5) $$\cos\beta_{1}=\frac{l_{1}^{2}+r^{2}-d_{1}^{2}}{2l_{1}r}$$

(6) $$\cos\beta_{2}=\frac{l_{2}^{2}+r^{2}-d_{2}^{2}}{2l_{2}r}$$

(7) $$l_{1}^{2}=r^{2}+d_{1}^{2}-2rd_{1}\cos\left(\pi -\alpha \right)$$

(8) $$l_{2}^{2}=r^{2}+d_{2}^{2}-2rd_{2}\cos\alpha$$

Substituting Equations (5)–(8) into Equation (4) yields:

(9) $$B_{Q}^{\prime }(t)=\frac{\mu i_{2}(t)}{2\pi }\frac{r-d_{2}\cos\alpha }{r^{2}+d_{2}^{2}-2rd_{2}\cos\alpha }-\frac{\mu i_{1}(t)}{2\pi }\frac{r+d_{1}\cos\alpha }{r^{2}+d_{1}^{2}+2rd_{1}\cos\alpha }$$

Since $i_{1}(t)$ and $i_{2}(t)$ are equal in magnitude and opposite in direction, let $i_{1}(t)=i_{2}(t)=i(t)$, then Equation (9) can be rewritten as:

(10) $$B_{Q}^{\prime }(t)=\frac{\mu i(t)}{2\pi }\left(\frac{r-d_{2}\cos\alpha }{r^{2}+d_{2}^{2}-2rd_{2}\cos\alpha }-\frac{r+d_{1}\cos\alpha }{r^{2}+d_{1}^{2}+2rd_{1}\cos\alpha }\right)$$

As can be seen from Equation (10), the magnetic flux densities generated at the point *Q* by the primary conductors flowing through the opposite currents largely cancel each other out. If the two conductors are completely coincident, the magnetic field generated at point *Q* will completely cancel out. However, the wires are physical entities and cannot completely overlap, so there is a small magnetic flux density at point *Q*. Through the differential principle of magnetic flux, it is ensured that the magnetic flux density in the magnetic core is relatively small, and the magnetic core is in a linear working area.

### 2.2. Linear Working Area of Ferrite

**Figure 5:**
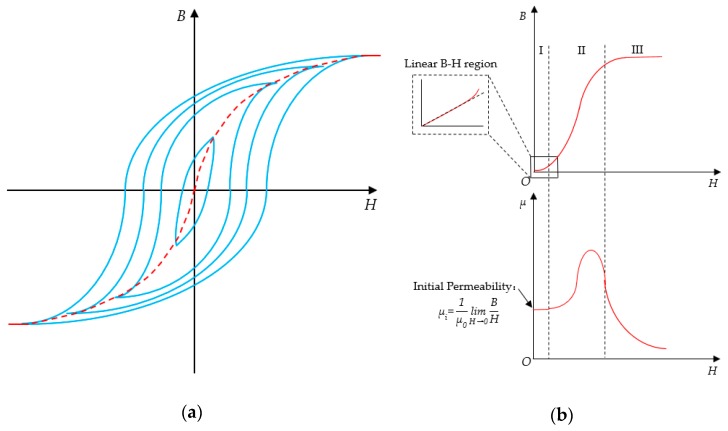
Magnetic characteristic curves: (**a**) high-frequency magnetization curves; (**b**) initial magnetization curve.

Ferrite is mostly used in the high-frequency field, so the focus is the characteristics of ferrite under high-frequency alternating magnetic fields. If an alternating magnetic field is applied to the ferrite core and the magnitude of the exciting magnetic field is changed, the high-frequency magnetization curve of the magnetic core material, as shown in Figure 5a, can be drawn. The magnetization curve from zero magnetization to saturation magnetization is usually called the initial magnetization curve of materials. The dashed line in Figure 5a is the initial magnetization curve of the material.

The initial magnetization curve of the ferrite material is shown in Figure 5b. The B–H relationship can be divided into three regions:

1. Linear B–H region at a low-magnetic flux density;
2. Non-linear B–H region at a medium magnetic flux density;
3. Highly non-linear B–H region at a high-magnetic flux density (saturated B–H region).

As can be seen from Figure 5b, the magnetic permeability of the ferrite material is non-linear and varies with the strength of the magnetic field. Core materials at different working magnetic densities have different applications. The high magnetic permeability of the core material and the stability of the magnetic permeability are the performance requirements of the sensor. Therefore, the ferrite core must work under a small magnetic field, and its magnetic permeability can be replaced by the initial permeability. The initial permeability is defined as the ratio of B to H when the magnetic field is near zero.

### 2.3. Mutual Inductance

**Figure 6:**
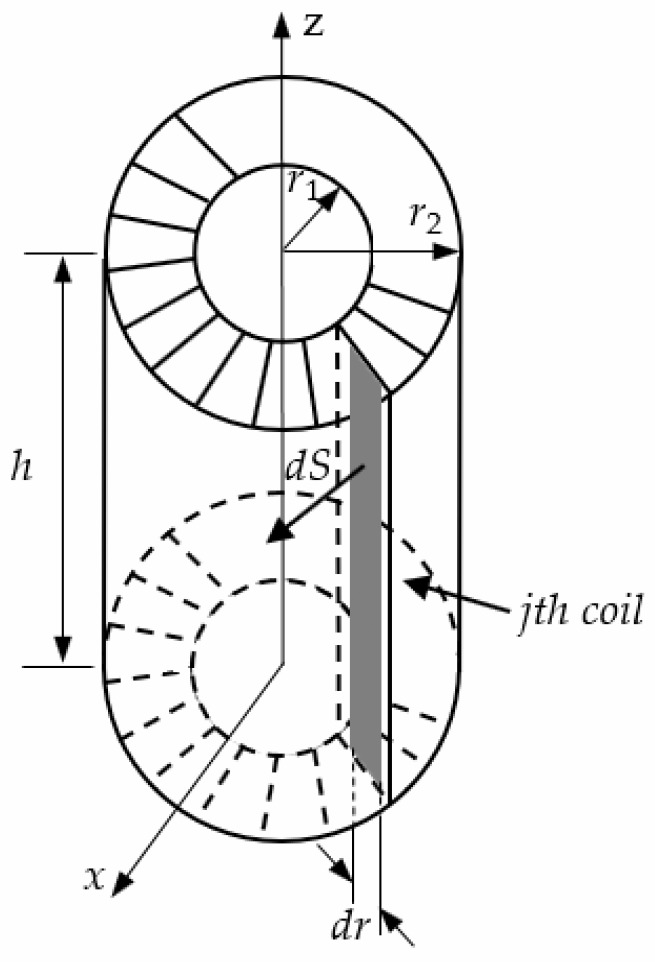
Analysis of the magnetic flux of a single coil.

The magnetic flux of a coil is shown in Figure 6. Taking a facet on the section surrounded by the jth turn of the figure, the magnetic flux flowing through the facet is:

(11) $$d\varphi_{j}=B_{Q}^{\prime }(t)dS=B_{Q}^{\prime }(t)hdr$$

where j=1~N. The magnetic flux flowing through the section surrounded by the jth coil is:

(12) $$\varphi_{j}=\int d\varphi_{j}=\int_{r_{1}}^{r_{2}}B_{Q}^{\prime }(t)hdr=\frac{\mu hi(t)}{4\pi }\left(\ln\frac{r_{2}^{2}+d_{2}^{2}-2r_{2}d_{2}\cos\alpha }{r_{1}^{2}+d_{2}^{2}-2r_{1}d_{2}\cos\alpha }-\ln\frac{r_{2}^{2}+d_{1}^{2}+2r_{2}d_{1}\cos\alpha }{r_{1}^{2}+d_{1}^{2}+2r_{1}d_{1}\cos\alpha }\right)$$

To simplify the formula, define three variables. $r_{2}/r_{1}$ is defined as the radius ratio k, $d_{2}/r_{1}$ is defined as the eccentricity m of conductor 2, and $d_{1}/r_{1}$ is defined as the eccentricity n of conductor 1. Then Equation (12) can be rewritten as:

(13) $$\varphi_{j}=\frac{\mu hi(t)}{4\pi }\left(\ln\frac{k^{2}+m^{2}-2km\cos\alpha }{1+m^{2}-2m\cos\alpha }-\ln\frac{k^{2}+n^{2}+2kn\cos\alpha }{1+n^{2}+2n\cos\alpha }\right)$$

Since the jth coil is interlinked once with the current, the flux linkage ($\psi_{j}$) of the section surrounded by the jth is equal to the magnetic flux ($\varphi_{j}$). The mutual inductance between the primary conductor and the jth coil is:

(14) $$M_{j}=\frac{\psi_{j}}{i(t)}$$

The total flux linkage of the N coils is:

(15) $$\psi =\sum_{j=1}^{N}\psi_{j}=\sum_{j=1}^{N}\varphi_{j}$$

**Figure 7:**
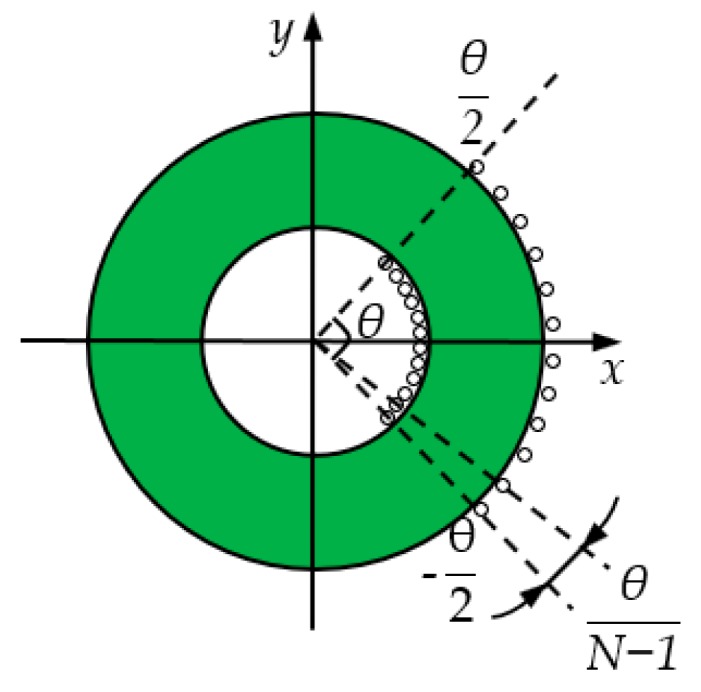
Analysis of the sensor coil’s distribution.

Since each coil is connected in series, the mutual inductance M of the sensor is the sum of the mutual inductance $M_{j}$ between each coil and the primary conductor.

(16) $$M=\frac{\psi }{i(t)}=\frac{\sum_{j=1}^{N}\psi_{j}}{i(t)}=\sum_{j=1}^{N}M_{j}$$

when considering that N coils are uniformly wound in the range of angle (−θ/2, θ/2), as shown in Figure 7. 

The angle corresponding to each coil was α=−θ/2+(j−1)θ/(N−1), where j=1~N. Then Equation (16) can be expressed as:

(17) $$M=\frac{\mu h}{4\pi }\sum_{j=1}^{N}\left(\ln\frac{k^{2}+m^{2}-2km\cos\left(-\frac{\theta }{2}+\frac{\left(j-1\right)\theta }{N-1}\right)}{1+m^{2}-2m\cos\left(-\frac{\theta }{2}+\frac{\left(j-1\right)\theta }{N-1}\right)}-\ln\frac{k^{2}+n^{2}+2kn\cos\left(-\frac{\theta }{2}+\frac{\left(j-1\right)\theta }{N-1}\right)}{1+n^{2}+2n\cos\left(-\frac{\theta }{2}+\frac{\left(j-1\right)\theta }{N-1}\right)}\right)$$

Equation (17) is established only when the coil enclosing angle is $0^{{}^{\circ}}<\theta <360^{{}^{\circ}}$. When $\theta =0^{{}^{\circ}}$ or $\theta =360^{{}^{\circ}}$, M=0.

Explanation 1: $\theta =0^{{}^{\circ}}$ is equivalent to the core without a coil, and the electromagnetic induction phenomenon does not exist, which means that the mutual inductance is zero.

Explanation 2: when $\theta =360^{{}^{\circ}}$, Equation (17) should be rewritten as:

(18) $$M=\frac{\mu h}{4\pi }\sum_{j=1}^{N}\left(\ln\frac{k^{2}+m^{2}-2km\cos\left(-\frac{\theta }{2}+\frac{\left(j-1\right)\theta }{N}\right)}{1+m^{2}-2m\cos\left(-\frac{\theta }{2}+\frac{\left(j-1\right)\theta }{N}\right)}-\ln\frac{k^{2}+n^{2}+2kn\cos\left(-\frac{\theta }{2}+\frac{\left(j-1\right)\theta }{N}\right)}{1+n^{2}+2n\cos\left(-\frac{\theta }{2}+\frac{\left(j-1\right)\theta }{N}\right)}\right)$$

no matter how other parameters change, the mutual inductance is always zero. Since the secondary windings evenly wrap around the entire core, the eccentricity of the current carrying conductor does not affect the output. For primary conductor 1, the mutual inductance between it and the secondary winding is $M_{1}$. For primary conductor 2, the mutual inductance between it and the secondary winding is $M_{2}$. Since the currents on the current-carrying conductors are equal in magnitude and opposite in direction, $M_{1}=-M_{2}$, the total mutual inductance is $M=M_{1}+M_{2}=0$.

## 3. Equivalent Circuit and Transmission Characteristics of the D-HFCT Sensor

### 3.1. Equivalent Circuit

**Figure 8:**
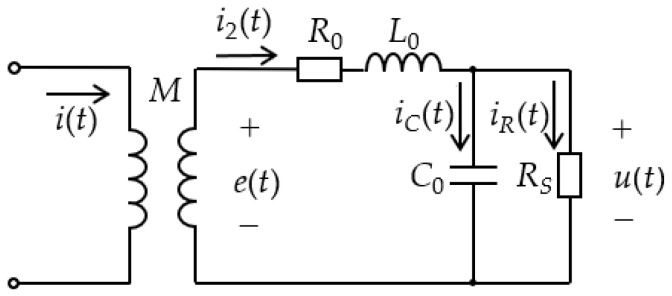
Time domain equivalent circuit.

The equivalent circuit of the D-HFCT sensor is shown in Figure 8. $R_{0}$, $L_{0}$, and $C_{0}$ are the internal resistance, self-inductance, and stray capacitance of the sensor, respectively. i(t) is the measured current flowing through the primary conductors. $i_{2}(t)$ is the induced current flowing through the secondary windings. M is the mutual inductance. e(t) is the induced voltage. $R_{S}$ is the sampling resistor connected to the output terminals. $i_{C}(t)$ and $i_{R}(t)$ are currents flowing through $C_{0}$ and $R_{S}$, respectively. u(t) is the sampling voltage across the sampling resistor.

According to Kirchhoff’s law, the following expression can be derived from Figure 8:

(19) $$e(t)=M\frac{di(t)}{dt}$$

(20) $$e(t)-R_{0}i_{2}(t)-L_{0}\frac{di_{2}(t)}{dt}-u(t)=0$$

(21) $$i_{2}(t)=C_{0}\frac{du(t)}{dt}+\frac{u(t)}{R_{S}}$$

The ferrite core makes the sensor’s self-inductance larger, and the current flowing through the stray capacitance is much smaller than that flowing through the sampling resistor. Therefore, the sensor’s self-integration conditions (22) and (23) are easily satisfied.

(22) $$L_{0}\frac{di_{2}\left(t\right)}{dt}>>R_{0}i_{2}\left(t\right)+u\left(t\right)$$

(23) $$\frac{u\left(t\right)}{R_{S}}>>C_{0}\frac{du\left(t\right)}{dt}$$

Simplify Equations (19)–(23) to obtain Equation (24):

(24) $$u\left(t\right)=\frac{MR_{S}}{L_{0}}i\left(t\right)$$

The output voltage collected by the sampling resistor is proportional to the current being measured, which is the same as the principle of measuring current with a shunt or a voltage divider resistor. It can also be seen from Equation (24) that the output voltage and the measured current are the same in frequency, but this does not mean that the frequency of the measured current can be arbitrary.

First, the sensor senses the measured current by the law of electromagnetic induction, so the measured current must be an alternating current, not a direct current. Secondly, the magnetization characteristics of the high-frequency magnetic material limit the allowable frequency band of the current to be measured. Finally, the actual operating frequency band is determined by the frequency response of the sensor.

### 3.2. Frequency Response

The most important characteristic of the D-HFCT sensor is the frequency response. The most important parameters are the cutoff frequency, bandwidth, and sensitivity of the sensor. These parameters depend on the structural parameters of the coil, and the specific parameter values of the sensor can be obtained through actual measurement.

Simplify Equations (19)–(21) to obtain Equation (25):

(25) $$M\frac{di(t)}{dt}=L_{0}C_{0}\frac{d^{2}u(t)}{dt^{2}}+\left(\frac{L_{0}}{R_{S}}+R_{0}C_{0}\right)\frac{du(t)}{dt}+\left(1+\frac{R_{0}}{R_{S}}\right)u(t)$$

**Figure 9:**
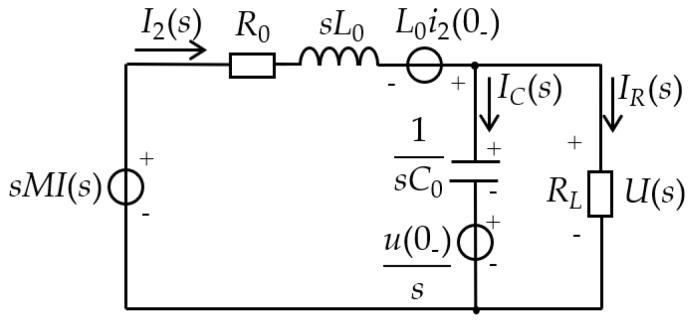
*S* domain equivalent circuit.

In order to derive the transfer function of the entire sensor measurement system, the time domain model of Figure 8 needs to be converted into the *S* domain model of Figure 9 by means of the Laplace transform method. Performing a Laplace transform on Equation (25) yields:

(26) $$M\left(sI\left(s\right)-i\left(0_{-}\right)\right)=L_{0}C_{0}\left(s^{2}U\left(s\right)-su\left(0_{-}\right)-u^{\prime }\left(0_{-}\right)\right)+\left(\frac{L_{0}}{R_{S}}+R_{0}C_{0}\right)\left(sU\left(s\right)-u\left(0_{-}\right)\right)+\left(1+\frac{R_{0}}{R_{S}}\right)U\left(s\right)$$

Considering that the initial state of the system is zero, Equation (26) can be simplified to:

(27) $$sMI\left(s\right)=\left(L_{0}C_{0}s^{2}+\left(\frac{L_{0}}{R_{S}}+R_{0}C_{0}\right)s+\left(1+\frac{R_{0}}{R_{S}}\right)\right)U\left(s\right)$$

The transfer function of the D-HFCT sensor is:

(28) $$H\left(s\right)=\frac{U\left(s\right)}{I\left(s\right)}=\frac{sM}{L_{0}C_{0}s^{2}+\left(\frac{L_{0}}{R_{S}}+R_{0}C_{0}\right)s+\left(1+\frac{R_{0}}{R_{S}}\right)}$$

According to the filter circuit, the transfer function of the second-order filter circuit is:

(29) $$A_{u}\left(s\right)=\frac{a_{0}+a_{1}s+a_{2}s^{2}}{b_{0}+b_{1}s+b_{2}s^{2}}$$

when $a_{1}=a_{2}=0$, the circuit is a second-order low-pass filter; when $a_{0}=a_{1}=0$, the circuit is a second-order high-pass filter; when $a_{0}=a_{2}=0$, the circuit is a second-order band-pass filter; when $a_{1}=0$, the circuit is a second-order band-stop filter. Therefore, one can determine that Equation (28) is a second-order band-pass filter circuit.

Taking the s=jω in Equation (28), the amplitude-frequency response can be obtained as follows:

(30) $$H\left(j\omega \right)=\frac{MR_{S}}{L_{0}+R_{0}C_{0}R_{S}}\frac{1}{1+j\left(\frac{\omega L_{0}C_{0}R_{S}}{L_{0}+R_{0}C_{0}R_{S}}-\frac{R_{S}+R_{0}}{\omega \left(L_{0}+R_{0}C_{0}R_{S}\right)}\right)}$$

(31) $$\left|H\left(j\omega \right)\right|=\frac{MR_{S}}{L_{0}+R_{0}C_{0}R_{S}}\frac{1}{\sqrt{1+{\left(\frac{\omega L_{0}C_{0}R_{S}}{L_{0}+R_{0}C_{0}R_{S}}-\frac{R_{S}+R_{0}}{\omega \left(L_{0}+R_{0}C_{0}R_{S}\right)}\right)}^{2}}}$$

Analysis 1: When $\omega_{r}=\sqrt{\frac{R_{S}+R_{0}}{L_{0}C_{0}R_{S}}}$, the corresponding resonant frequency and sensitivity are:

(32) $$f_{r}=\frac{1}{2\pi }\sqrt{\frac{R_{S}+R_{0}}{L_{0}C_{0}R_{S}}}$$

(33) $${\left|H\left(j\omega \right)\right|}_{r}={\left|H\left(j\omega \right)\right|}_{\max}=\frac{MR_{S}}{L_{0}+R_{0}C_{0}R_{S}}$$

Analysis 2: When $\omega_{h}=\frac{L_{0}+R_{0}C_{0}R_{S}}{L_{0}C_{0}R_{S}}$ or $\omega_{l}=\frac{R_{S}+R_{0}}{L_{0}+R_{0}C_{0}R_{S}}$, the corresponding amplitude-frequency responses are:

(34) $${\left|H\left(j\omega \right)\right|}_{h}=\frac{1}{\sqrt{2}}{\left|H\left(j\omega \right)\right|}_{\max}$$

(35) $$20\mathrm{lg}{\left|H\left(j\omega \right)\right|}_{h}=20\mathrm{lg}{\left|H\left(j\omega \right)\right|}_{\max}-20\mathrm{lg}\sqrt{2}=20\mathrm{lg}{\left|H\left(j\omega \right)\right|}_{\max}-3dB$$

(36) $${\left|H\left(j\omega \right)\right|}_{l}=\frac{1}{\sqrt{2}}{\left|H\left(j\omega \right)\right|}_{\max}$$

(37) $$20\mathrm{lg}{\left|H\left(j\omega \right)\right|}_{l}=20\mathrm{lg}{\left|H\left(j\omega \right)\right|}_{\max}-20\mathrm{lg}\sqrt{2}=20\mathrm{lg}{\left|H\left(j\omega \right)\right|}_{\max}-3dB$$

according to the −3 dB principle, the upper cutoff frequency, lower cutoff frequency, and bandwidth of the sensor can be determined.

(38) $$f_{h}=\frac{1}{2\pi }\frac{L_{0}+R_{0}C_{0}R_{S}}{L_{0}C_{0}R_{S}}$$

(39) $$f_{l}=\frac{1}{2\pi }\frac{R_{S}+R_{0}}{L_{0}+R_{0}C_{0}R_{S}}$$

(40) $$f_{bw}=f_{h}-f_{l}=\frac{1}{2\pi }\left(\frac{L_{0}+R_{0}C_{0}R_{S}}{L_{0}C_{0}R_{S}}-\frac{R_{S}+R_{0}}{L_{0}+R_{0}C_{0}R_{S}}\right)$$

It can be seen from Equations (32),(33), and (38)–(40) that the upper cutoff frequency, lower cutoff frequency, bandwidth and sensitivity of the sensor are determined by the coil parameters $R_{0}$, $L_{0}$, and $C_{0}$ and the sampling resistor $R_{S}$ of the sensor. Select the appropriate coil parameters and sampling resistor to adjust the frequency response of the sensor.

### 3.3. Influence of Conductor Eccentricity and Coil Parameters on Sensitivity

Equation (24) gives the output response of the sensor. The output voltage is proportional to the measured current, and the proportional coefficient (sensitivity) is approximately the ratio of the product of the mutual inductance and the sampling resistance to the self-inductance. Equation (24) can be rewritten as:

(41) $$u\left(t\right)=\frac{R_{S}}{2N^{2}\ln k}\sum_{j=1}^{N}\left(\ln\frac{k^{2}+m^{2}-2km\cos\left(-\frac{\theta }{2}+\frac{\left(j-1\right)\theta }{N-1}\right)}{1+m^{2}-2m\cos\left(-\frac{\theta }{2}+\frac{\left(j-1\right)\theta }{N-1}\right)}-\ln\frac{k^{2}+n^{2}+2kn\cos\left(-\frac{\theta }{2}+\frac{\left(j-1\right)\theta }{N-1}\right)}{1+n^{2}+2n\cos\left(-\frac{\theta }{2}+\frac{\left(j-1\right)\theta }{N-1}\right)}\right)i\left(t\right)$$

According to Equation (41), the factors affecting the sensitivity of the sensor include: N (the coil turns of the secondary windings); θ (the angle occupied by the secondary windings); m and n (the eccentricities of the primary conductors); $r_{1}$ and $r_{2}$ (inner radius and outer radius of the core); h (height of the core); $\mu_{r}$ (relative permeability of the core); $R_{S}$ (sampling resistor).

This section used Matrix Laboratory to simulate and analyze the influence of the eccentricities (m, n) of the conductors and the structural parameters (N, θ) of the coil on the sensitivity of the sensor when the core parameters (k, h, $\mu_{r}$) and sampling resistor were fixed.

The parameters selected for the simulation were as follows: N=50, $\theta =180^{{}^{\circ}}$, m=0.5, n=0.5, k=2, h=5, $\mu_{r}=100$, $R_{S}=1\Omega$, and the above geometrical dimensions were in millimeters. In the following analysis, when one of the parameters changed, the other parameters kept the above values unchanged.

#### 3.3.1. The Number of Turns of the Secondary Windings

The number of turns of the coil directly affected the total flux linkage of the coil, thus affecting the mutual inductance and self-inductance of the coil. The following values were taken as the value of N: 10, 20, 30, 40, 50, 60, 70, 80, 90, 100. The corresponding sensitivities are shown in Table 1.

When N increased and θ did not change (the coil density increased), the sensitivity of the sensor gradually decreased, where coil density was the ratio of N−1 to θ.

**Figure 10:**
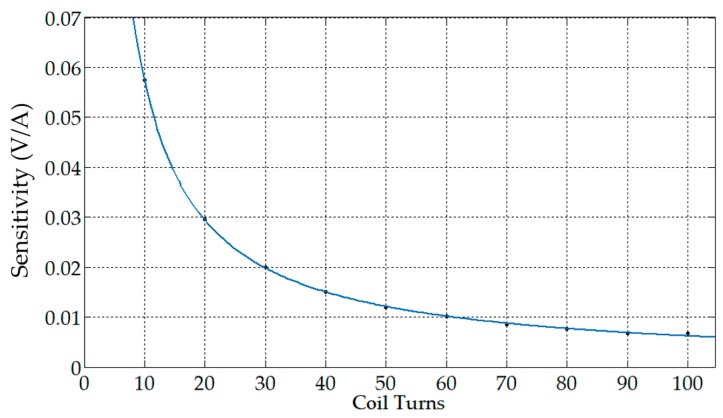
The relationship between the number of turns and sensitivity.

The functional relationship derived from the power function approximation method based on the above calculation results was: $y=0.5341x^{-0.9676}$. The corresponding fitting curve is shown in Figure 10. It can be seen that the relationship between the number of turns of the coil N and the sensitivity of the sensor was close to the inverse proportional function. As the number of turns of the coil increased, the corresponding sensitivity became smaller, and the response of the output also became smaller. The reason for exhibiting the above characteristics was that the increase in the number of turns of the coil had a greater influence on the self-inductance than on the mutual inductance. The self-inductance was proportional to the square of the turns of the coil, and the mutual inductance was proportional to the number of turns of the coil. It is worth noting that reducing the number of turns can increase the output response, but a decrease in the number of turns can cause a sharp decrease in the sensor’s self-inductance. Since the premise of Equation (24) is Equation (22), the sensor’s self-inductance is reduced to a certain extent, and Equation (22) will not be satisfied, which means that the number of turns cannot be chosen too small.

#### 3.3.2. Distribution Angle of the Secondary Windings

The effect of the angle θ occupied by the secondary windings on the sensitivity is shown in Table 2, and the step of the angle changes was $30^{{}^{\circ}}$. When θ increased and N did not change, the coil density and the sensitivity decreased.

**Figure 11:**
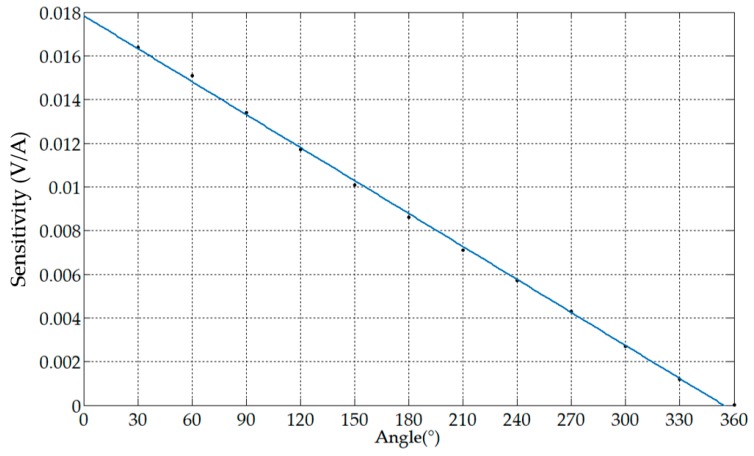
The relationship between distribution angle and sensitivity.

The functional relationship obtained by least squares fitting based on the above results was: $y=-5.036\times 10^{-5}x+0.01785$. The corresponding fitting curve is shown in Figure 11. The angle occupied by the secondary winding was inversely related to the sensitivity of the sensor. In order to ensure that the output response of the sensor was large enough, the angle θ should be small, but it should be noted that the angle θ should not be extremely small. The closer the coil distance, the larger the inter-turn capacitance of the sensor, and the smaller the upper cutoff frequency of the sensor.

**Figure 12:**
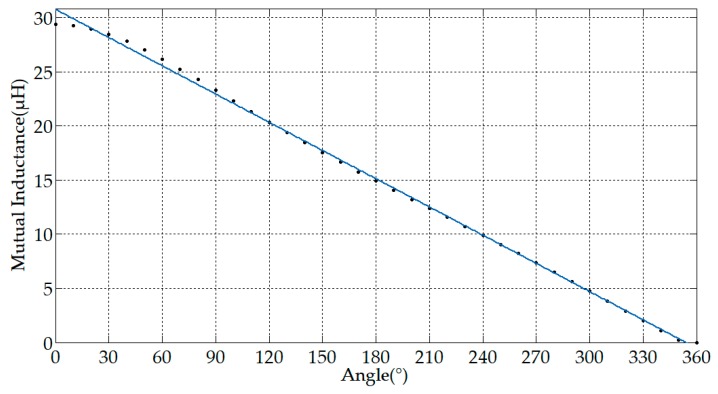
The relationship between distribution angle and mutual inductance.

It can be seen from Equations (24) and (41) that the distribution angle of the secondary windings is only related to the mutual inductance of the sensor. The relationship between the coil distribution angle and the sensitivity can also be regarded as the relationship between the coil distribution angle and the mutual inductance, because the self-inductance did not change with the coil distribution angle. The effect of the angle θ occupied by the secondary windings on the mutual inductance is shown in Figure 12, and the step of the angle changes is $10^{{}^{\circ}}$.

#### 3.3.3. Eccentricities of the Primary Conductors

**Figure 13:**
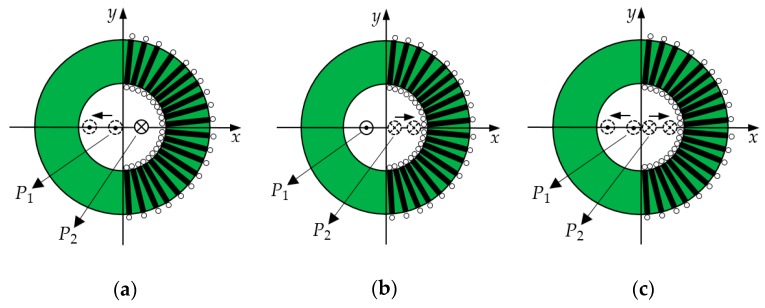
Schematic diagrams of eccentricity changes: (**a**) Case 1; (**b**) Case 2; and (**c**) Case 3.

The previous discussions assumed that the eccentricities of the two primary conductors were the same. In this section, the three cases shown in Figure 13 will be analyzed.

Case 1: The primary conductor 2 was fixed (eccentricity m=0.5), the eccentricity of primary conductor 1 changed from n=0.1 to n=0.9, and the step of eccentricity change was 0.1.

Case 2: The primary conductor 1 was fixed (eccentricity n=0.5), the eccentricity of primary conductor 2 changed from m=0.1 to m=0.9, and the step of eccentricity change was 0.1.

Case 3: The eccentricities of the primary conductors 1 and 2 changed from 0.1 to 0.9 at the same time, and the step size of the change was 0.1.

The eccentricity of the primary conductor was independent of the sensor’s self-inductance and only affected the mutual inductance of the sensor. According to Equation (24), the influence of the eccentricity of the primary conductor on the mutual inductance was the same as its influence on the sensitivity.

The mutual inductance values in the above cases are given in Table 3.

As can be seen from the above table data, the positions of the primary conductors had an effect on the mutual inductance of the sensor, which is the sacrifice one has to make in order to make the core work in the linear working area. It can be seen from the calculation data that when the eccentricity of the primary conductor increased, the residual magnetic field of the two differential primary conductors gradually increased, and the corresponding mutual inductance gradually increased. A large eccentricity was needed to achieve greater mutual inductance and sensitivity. It is worth considering that if the eccentricity of a conductor is too large, it means that the conductor is closer to the core, which will cause local saturation of the core and affect the normal measurement of the sensor. Therefore, in the subsequent experimental part, the eccentricity was selected at a medium level (m=0.5,n=0.5).

## 4. Experimental Setup and Results

### 4.1. The D-HFCT Sensor Design

**Figure 14:**
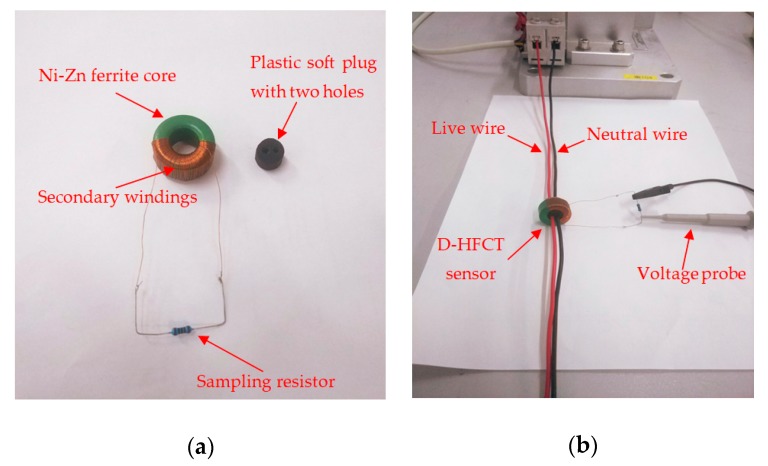
Composition and installation diagrams of the D-HFCT sensor. (**a**) Components of the D-HFCT sensor; (**b**) installation of the D-HFCT sensor.

Figure 14 shows the composition and installation diagrams of a D-HFCT sensor. The sensor matched the differential threading method. The live wire and neutral wire passed through the perforated plastic soft plug in the middle of the magnetic ring to ensure that the core was away from the non-linear working area. The voltage signal on the sampling resistor was collected by the voltage probe and displayed on the oscilloscope to determine if an arc fault had occurred by whether the waveform had a high-frequency voltage signal. The relevant magnetic core material parameters of the sensor are shown in Table 4, and the relevant electrical parameters are shown in Table 5.

To verify the sensitivity of the sensor, the circuit shown in Figure 14b was connected to an arbitrary waveform generator. The waveform generator was RIGOL-DG1022 (This is the model number of a device, RIGOL represents the manufacturer, and DG1022 represents the device type), and its maximum signal frequency was up to 20 MHz. This frequency met the experimental requirements. In the experiment, a 1 ohm resistor was connected in series in the circuit as the sampling resistor, and the voltage value on it was equal to the current value generated by the waveform generator. The sampling resistance of the D-HFCT sensor was 3.6 ohms. Because its self-resistance is relatively small, the sampling resistance at the output should not be too large. According to Equations (38) and (39), the sampling resistance increases, the upper cutoff frequency decreases, and the lower cutoff frequency increases, which causes the bandwidth of the sensor to decrease.

**Figure 15:**
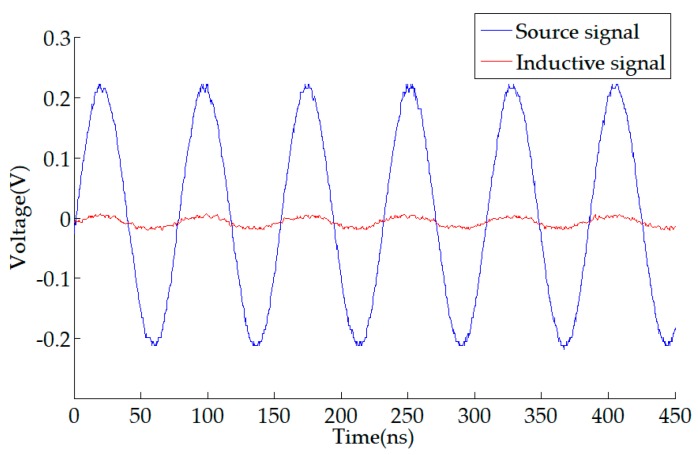
Output signal of the waveform generator and induced signal of the D-HFCT sensor.

The sensitivity of the sensor derived from the data in the table was 0.0387 V/A. The sensitivity of the D-HFCT sensor obtained from the measured waveform data of Figure 15 was 0.0444 V/A (0.01 V/0.225 A), and the accuracy was 0.86. Mutual inductance was the main reason why it affected the accuracy of the sensor. On the one hand, the mutual inductance value was calculated by the theoretical formula, not the actual measurement. The measurement of mutual inductance becomes difficult due to the presence of stray capacitance. On the other hand, the unevenness of the secondary windings caused a deviation between the actual mutual inductance value and the theoretically calculated mutual inductance value.

**Figure 16:**
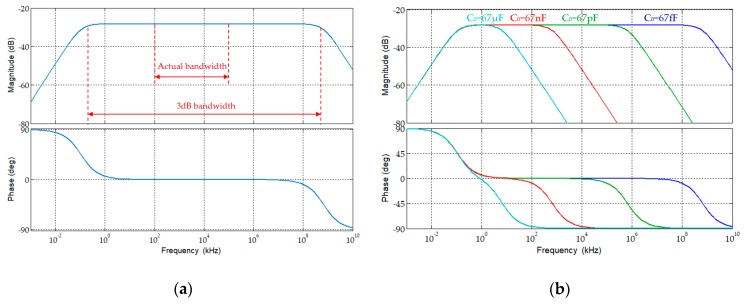
Frequency response of the D-HFCT sensor. (**a**) $C_{0}=67fF$; (**b**) $C_{0}$ increases.

Figure 16a shows a Bode diagram corresponding to the frequency response of the D-HFCT sensor, which was drawn in Matlab. The lower cutoff frequency of the sensor was several hundred Hz, and the upper cutoff frequency exceeded 10,000 MHz. This is an ideal 3 dB bandwidth, and the bandwidth was actually less than this value. Because the frequency of the stray capacitance and ground capacitance of the sensor was significantly increased in the frequency range above several megahertz, it was much larger than the data measured in Table 5. It can be seen from Equation (38) and Figure 16b that the upper cutoff frequency will drop sharply. In addition, the effective operating frequency of the selected high-frequency core was 0.1 MHz–100 MHz, and signals outside this band will be attenuated, which means that the actual frequency band was about 0.1 MHz–100 MHz.

### 4.2. Series Arc Fault Simulation Experiment System

**Figure 17:**
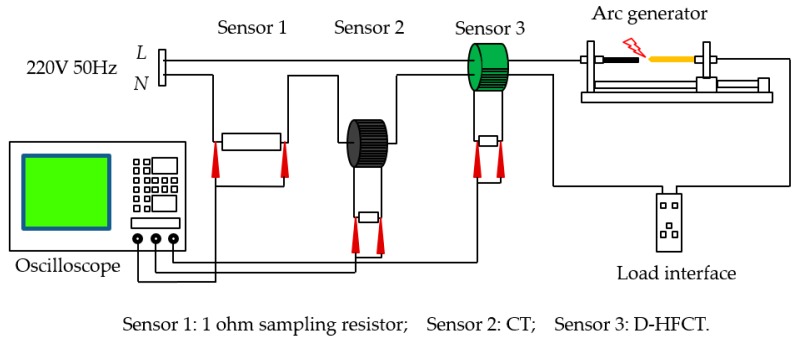
Schematic diagram of the series arc fault experimental simulation system.

**Figure 18:**
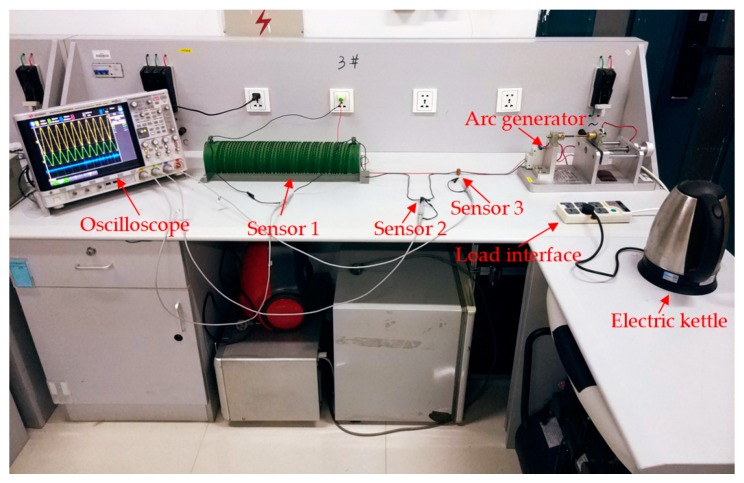
Physical diagram of the series arc fault experimental simulation system.

The series arc fault experiment simulation system was designed as shown in Figure 17 and Figure 18. The 220 V, 50 Hz AC power was connected between the live line and the neutral line. Three sensors were selected in the experiment: Sensor 1 was a 1 ohm power resistor rated at 5000 W for collecting the actual current signal. The voltage across the resistor was equal to the value of the current flowing. The purpose of setting Sensor 1 was to collect current signals of all frequencies and to compare the signal acquisition effects with that of CT and D-HFCT. For Sensor 2, the model was HCT215-5 A/2.5 mA, which is a common straight-through current transformer. It operated at 50 Hz and was used to acquire low-frequency current signals. In the experiment, the sampling resistance of Sensor 2 was 100 ohms. The acquired signal waveform was compared with that of Sensor 3 to show the effect of the D-HFCT sensor for fault arc detection. For Sensor 3, through the differential threading method of the primary conductors, the magnetic flux in the core of the high-frequency current transformer sensor was significantly reduced, and the core was operated in the linear working area, and a relatively complete high-frequency signal could be collected.

**Figure 19:**
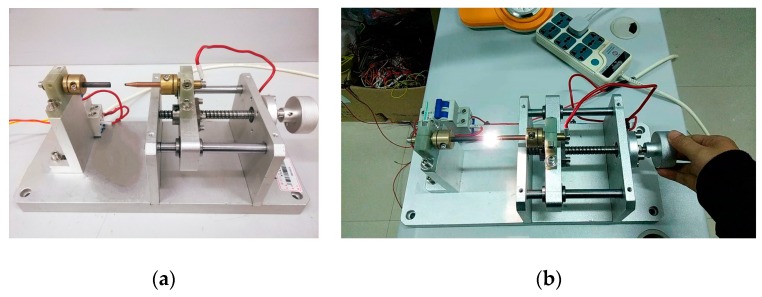
Arc generator. (**a**) Disconnected arc generator; (**b**) arc generator and its arc.

The arc generator shown in Figure 19 was designed according to standards IEC 62606-2017 [5] and UL 1699 [6]. It consisted of a fixed electrode and a movable electrode. When the two electrodes are separated, an arcing phenomenon will occur, which can be used to simulate an arc fault occurring in a series circuit. A variety of different loads commonly used by residents were connected in a series through the load interface. The model number of the oscilloscope was KEYSIGHT-DSOX4024A, and its maximum bandwidth was 200 MHz, which met our measurement needs. Three channels were selected to compare the waveform differences of the three sensors.

Through the load interface, the specified loads were connected into the circuit in turn. The initial working state was no arc state, no gap among the two poles of the arc generator, and the oscilloscope acquisition mode was set to the trigger mode. The arc generator was controlled to slowly separate the two poles, creating a blazing arc among the two poles. The arc signal caused the oscilloscope to trigger and stop. The oscilloscope displays the analog signals acquired by the three channels over a certain period of time.

### 4.3. Single Load Experiments

**Figure 20:**
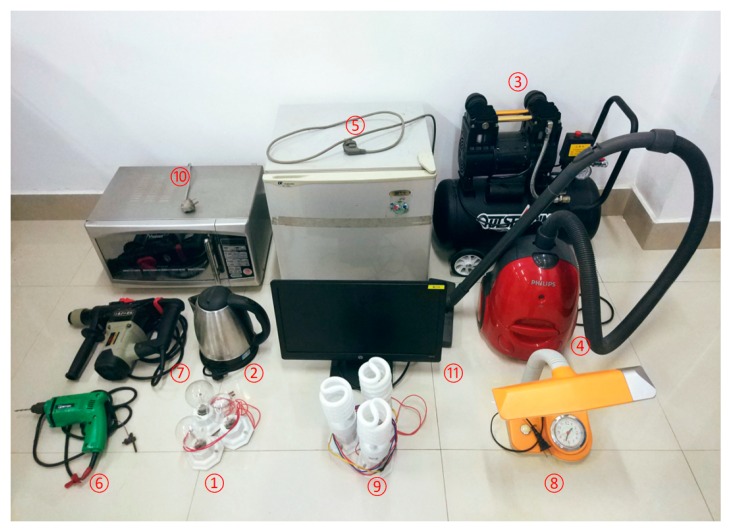
Physical diagram of experimental loads.

Three different types of loads were used in the experiment: resistive load (three incandescent lamps and one electric kettle), inductive load (one air compressor, one vacuum cleaner, one refrigerator, one electric drill and one electric hammer), and non-linear load (one fluorescent lamp, three energy-saving lamps, a microwave oven, and a computer screen). The physical diagram is shown in Figure 20 and information about the loads in Figure 20 is shown in Table 6.

#### 4.3.1. Resistive Load Experiments

**Figure 21:**
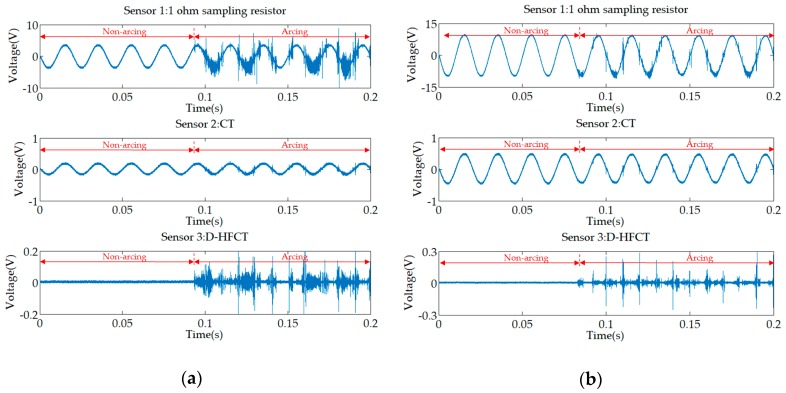
Experimental waveforms of resistive loads. (**a**) Incandescent lamp; (**b**) electric kettle.

The working waveforms of incandescent lamps and electric kettles are shown in Figure 21. It is worth noting that the output signal of the experimental part was a voltage signal, which was proportional to the current to be measured. The voltage waveform and the measured current waveform had the same shape but differed in amplitude by a proportional constant. In the case of no misunderstanding, the output voltage signal was called current signal.

The two output waveforms of the 1 ohm sampling resistor reflected the current changes in the non-arc and arc states, which contained the components of the various frequencies of the current. In the normal state (before 0.1 s), an incandescent lamp and electric kettle were close to the linear resistors with constant resistance, and their current waveforms were standard, undistorted sine waves. The magnitude of the waveform depends on their resistance. When an arc fault occurs, the current signal is superimposed with a large amount of high-frequency noise. 

The low-frequency sensor CT collected the low-frequency components of the current. The comparison of the non-arc state and the arc state of the low-frequency signal showed that the low-frequency waveform changed little and was not affected by the arc. The flat shoulder feature and the zero-time characteristic of the zero-crossing mentioned in many studies become inconspicuous due to the appearance of a high-frequency signal. This indicates that it is difficult to detect arc faults from the low-frequency components of the current signal for resistive loads.

The high-frequency sensor D-HFCT collected the high-frequency components of the current. In the normal state, the frequency of the current signal was approximately 50 Hz, and the output of the D-HFCT sensor was close to zero. In the arc state, the high-frequency components of the current signals can be coupled by the D-HFCT sensor. There was a clear difference between the normal state and the fault state.

#### 4.3.2. Inductive Load Experiments

The inductive load was not a purely inductive load, but its inductance was much larger than the resistance and it exhibited almost a pure inductor. Common inductive loads are mainly loads containing electric motors, such as the air compressor, vacuum cleaner, refrigerator, electric drill, and electric hammer used in this experiment. Of course, electric fans and washing machines are also inductive loads.

**Figure 22:**
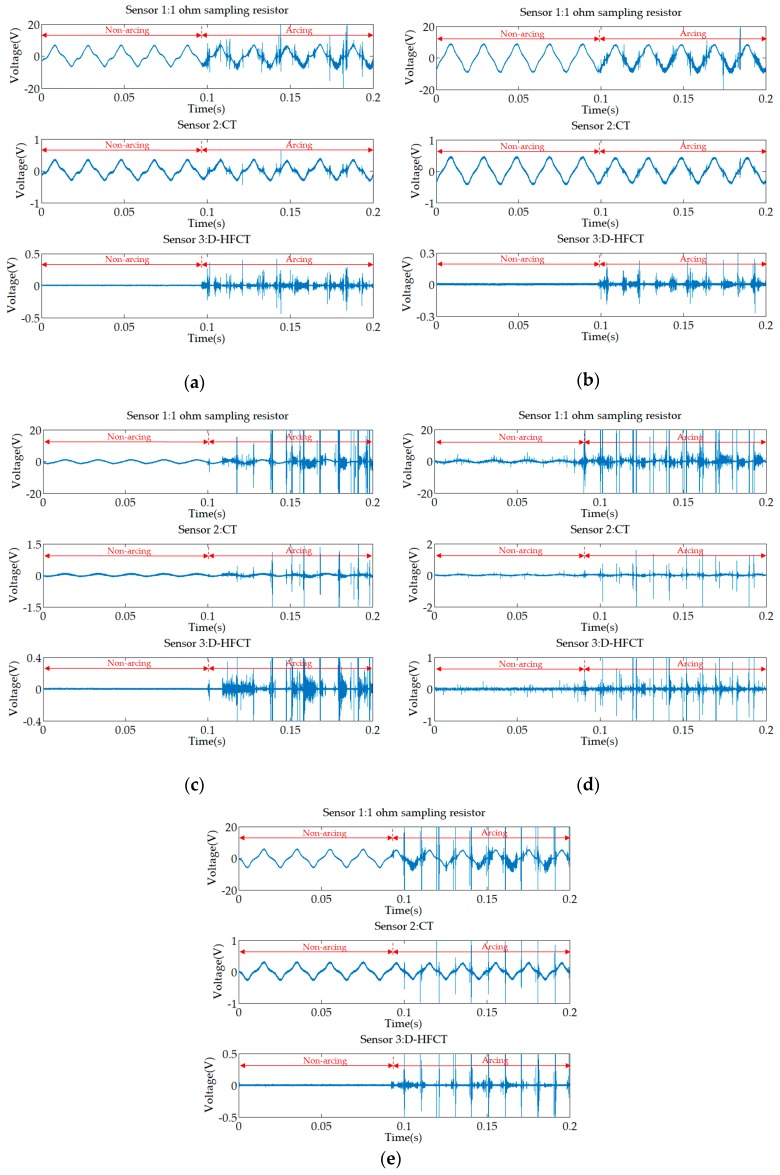
Experimental waveforms of inductive loads. (**a**) Air compressor; (**b**) vacuum cleaner; (**c**) refrigerator; (**d**) electric drill; (**e**) electric hammer.

Figure 22a–e shows experimental waveforms for the five inductive loads in Table 6. Comparing the five waveforms acquired from Sensor 1, before the arc occurred, unlike the previous resistive loads, the current waveforms were no longer standard undistorted sine waves, but sine waves with different distortion levels. When an arc fault occurs, the amplitude of the high-frequency noise of the inductive load is much larger than that of the resistive load, which also means that the arc burns extremely strongly.

For Sensor 2, the high-frequency noise of the air compressor (Figure 22a) and the vacuum cleaner (Figure 22b) was not obvious, and their amplitude was equivalent to the normal current amplitude, and the high-frequency component was significantly reduced, which easily leads to the occurrence of a missed judgment phenomenon. Although the high-frequency components of the refrigerator (Figure 22c), electric drill (Figure 22d), and electric hammer (Figure 22e) were attenuated, they can still distinguish between normal and fault conditions.

For Sensor 3, the comparison of the inductive load waveforms with the resistive load waveforms showed that the D-HFCT sensor was consistent in the fault current high-frequency signal acquisition and can help us to detect the arc fault.

#### 4.3.3. Non-Linear Load Experiments

There are many types of non-linear loads in our lives, such as soft starter, rectifier, inverter, fluorescent lamp, and dimming lamp. The main feature of the non-linear load was that the current waveform was non-sinusoidal. If only low-frequency signals of current are used for arc fault detection, this will lead to missed judgment and misjudgment of arc faults. For example, when a load such as a dimming lamp, an electric drill, or a switching power supply is working normally, a flat shoulder or zero-duration time with a current zero-crossing occurs, but for resistive loads, this is equivalent to an arc fault.

**Figure 23:**
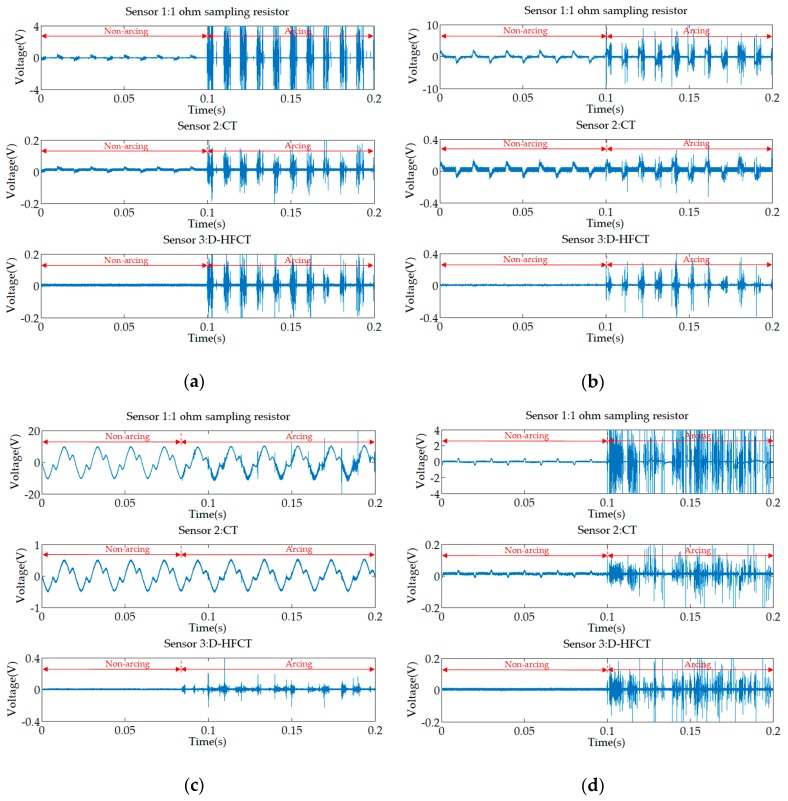
Experimental waveforms of non-linear loads. (**a**) Fluorescent lamp; (**b**) energy-saving lamp; (**c**) microwave oven; (**d**) computer screen.

Figure 23 shows experimental waveforms for a fluorescent, energy-saving lamp, microwave oven, and computer display. From the waveforms of Sensor 1, the distortion of the low-frequency current waveforms of the non-linear loads were more obvious than that of the inductive load. The waveforms of different types of loads and different working conditions vary greatly, and it is difficult to obtain a uniform feature to detect arc faults. However, it can be seen from the waveforms of Sensor 3 that, like the resistive load experiments and the inductive load experiments, the high-frequency burr characteristics of the arc were obvious.

For Sensor 2, CT was usually used to measure low-frequency signals. Comparing the waveforms of Sensor 2 in Figure 23a,b,d, it can be found that, in the case of low power load, some high-frequency noise can still be coupled. However, it can be found from several waveforms that the high-frequency signal coupled by the low-frequency transformer will have a glitch loss. In the case of high-power load (Figure 23c), the low-frequency transformer can hardly be coupled to the high-frequency signal.

### 4.4. High-Power Shielding Load Experiments

**Figure 24:**
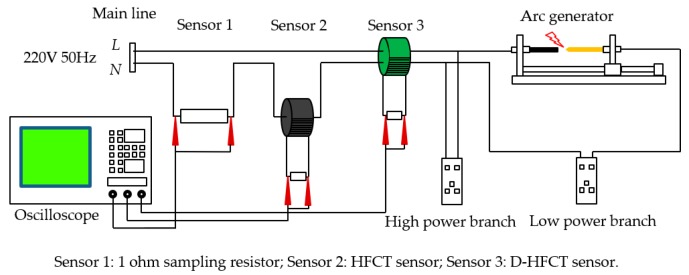
Schematic diagram of the high-power shielding load experiment.

**Figure 25:**
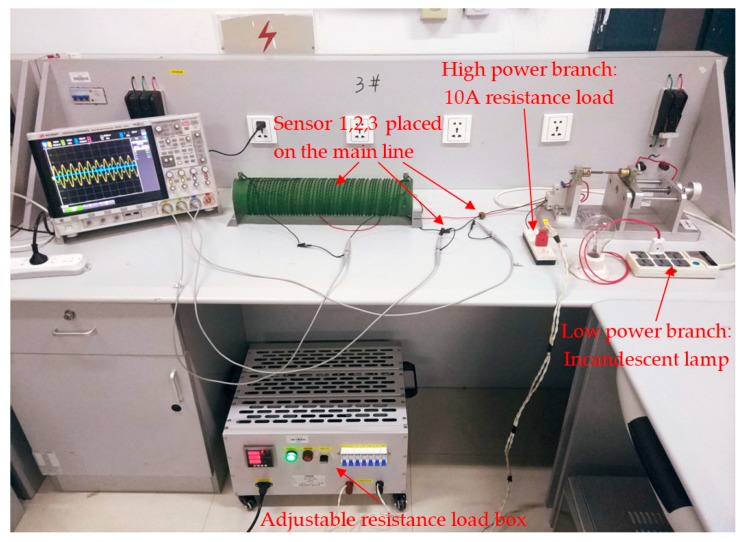
High-power shielding load experimental system.

The schematic diagram of the high-power shielding load experiment is shown in Figure 24. The arc simulation platform of the previous section was modified to connect a high-power load (high-power branch) in parallel with the arc fault branch (low-power branch). In order to compare the detection results, all sensors were placed on the main line. The actual circuit connection diagram is shown in Figure 25.

**Figure 26:**
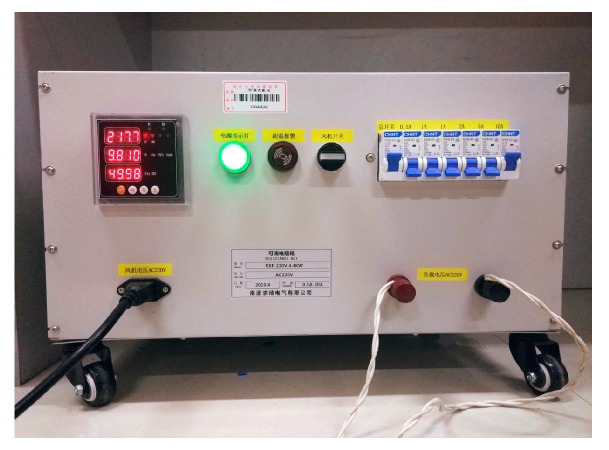
Adjustable resistance load box.

In the two sets of experiments, the low-power loads were a 200 W incandescent lamp and a 11 W fluorescent lamp. The high-power load was an adjustable resistor that was replaced with an adjustable resistor box. The physical diagram is shown in Figure 26. By controlling the on and off of different miniature circuit breakers, the resistance and the current level of the output can be adjusted. In the experiment, we always kept the resistance box output 10 A resistive current, which was equivalent to a high-power shielding load.

**Figure 27:**
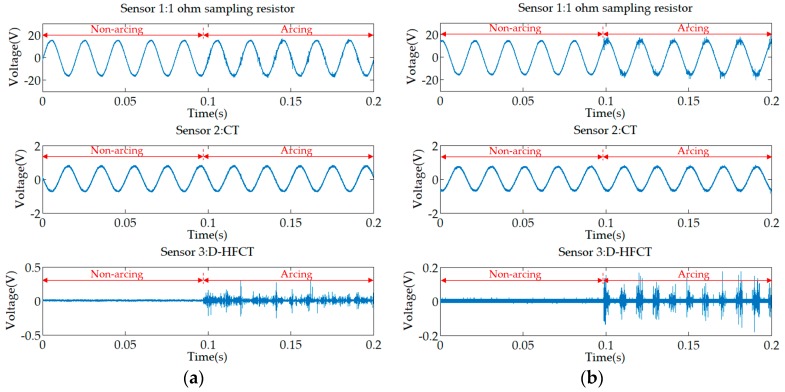
Experimental waveforms of the main line. (**a**) Incandescent lamp; (**b**) fluorescent lamp.

The waveforms of the two sets of experiments are shown in Figure 27. Compared to the normal state, the two waveforms of Sensor 2 were still undistorted sinusoidal waveforms in the arc state, that is, the arc fault cannot be detected. For the waveform of Sensor 1, the high-frequency noise of the incandescent lamp (Figure 21a) and the fluorescent lamp (Figure 23a) was extremely noticeable in the previous experiments. But when a low-power load was connected in parallel with a high-power load, the faulty high-frequency noise signal was almost submerged. This means that it was difficult to directly sample the current to detect this arc fault. For Sensor 3, similar to the previous experimental results, the difference between normal and fault states was obvious. In the normal state, the current cancellation of the differential threading method caused most of the current signal to be cancelled out, and the attenuation of the low-frequency signal by the ferrite material further attenuated the remaining normal current. In the fault state, the low-frequency normal high-current was eliminated, but the ferrite material could still extract the high-frequency noise of the small fault current.

## 5. Conclusions and Prospects

This paper presented a D-HFCT sensor for low-voltage series arc fault detection. Through the description of its working principle, analysis, and calculation of relevant parameters, a D-HFCT sensor was designed, and its sensitivity and working frequency band were verified. In order to test the effect of the sensor in arc fault detection, a series arc fault simulation experiment system was established. A variety of single load (11 types) arc fault detection experiments and high-power shielding load (2 types) arc fault detection experiments were carried out, and the signal acquisition effects of a 1 ohm power sampling resistor, CT, and D-HFCT were compared. The feasibility of D-HFCT in the series arc fault detection method based on high-frequency signal acquisition was confirmed.

The D-HFCT sensor and related experiments designed in this paper were carried out in a relatively ideal laboratory environment. Although the high-frequency signal of the D-HFCT sensor for arc fault detection was very effective for arc fault detection, there were still some problems that need to be studied in future work. First of all, the shielding problem of the sensor and the position fixing method of the primary conductor need to be studied in depth. Secondly, as the power electronic load is increasingly common in life, the interference problem of the switching power supply operating in the high-frequency state to the high-frequency signal of the arc fault also needs to be paid attention. Thirdly, the application of the sensor in the actual circuit should also be studied in future work.

## Figures and Tables

**Table 1:** Sensitivities corresponding to different coil turns.

Coil Turns	Distribution Angle (°)	Coil Density (1/°)	Sensitivity (Voltage/Ampere)
10	180	0.0550	0.0575
20	180	0.1056	0.0296
30	180	0.1611	0.0199
40	180	0.2167	0.0150
50	180	0.2722	0.0120
60	180	0.3278	0.0101
70	180	0.3833	0.0086
80	180	0.4389	0.0076
90	180	0.4944	0.0067
100	180	0.5500	0.0061

**Table 2:** Sensitivities corresponding to different distribution angles.

Coil Turns	Distribution Angle (°)	Coil Density (1/°)	Sensitivity (V/A)
50	30	1.6333	0.0164
50	60	0.8167	0.0151
50	90	0.5444	0.0134
50	120	0.4083	0.0117
50	150	0.3267	0.0101
50	180	0.2722	0.0086
50	210	0.2333	0.0071
50	240	0.2042	0.0057
50	270	0.1815	0.0043
50	300	0.1633	0.0027
50	330	0.1485	0.0012
50	360	0.1361	1.9516 × 10^−^^18^

**Table 3:** Mutual inductance values of different eccentricities.

Eccentricity	Mutual Inductance (μH)	Eccentricity	Mutual Inductance (μH)	Eccentricity	Mutual Inductance (μH)
m=0.5, n=0.1	8.9299	m=0.1, n=0.5	9.0849	m=0.1, n=0.1	3.1123
m=0.5, n=0.2	10.4791	m=0.2, n=0.5	10.6124	m=0.2, n=0.2	6.1891
m=0.5, n=0.3	12.0006	m=0.3, n=0.5	12.0992	m=0.3, n=0.3	9.1974
m=0.5, n=0.4	13.4791	m=0.4, n=0.5	13.5325	m=0.4, n=0.4	12.1092
m=0.5, n=0.5	14.9024	m=0.5, n=0.5	14.9024	m=0.5, n=0.5	14.9024
m=0.5, n=0.6	16.2615	m=0.6, n=0.5	16.2027	m=0.6, n=0.6	17.5618
m=0.5, n=0.7	17.5505	m=0.7, n=0.5	17.4298	m=0.7, n=0.7	20.0779
m=0.5, n=0.8	18.7665	m=0.8, n=0.5	18.5827	m=0.8, n=0.8	22.4468
m=0.5, n=0.9	19.9087	m=0.9, n=0.5	19.6623	m=0.9, n=0.9	24.6685

**Table 4:** Parameters of the Ni–Zn ferrite core.

Application Frequency (MHz)	Saturation Flux Density (T)	Initial Relative Permeability	Inner Diameter (mm)	Outer Diameter (mm)	Thickness (mm)
0.1–100	0.2	300	9.75	20.20	10.26

**Table 5:** Parameters of the D-HFCT sensor.

Parameters	Values	Parameters	Values
Wire diameter	0.15 mm	$R_{0}$	2.3 Ω
N	100	$L_{0}$	8.69 mH
θ	180°	$C_{0}$	67.229 fF
m	0.5	M	93.35 μH
n	0.5	$R_{S}$	3.6 Ω

**Table 6:** Parameters of different types of loads.

Load Number	Load	Load Type	Total Active Power (W)
1	Incandescent lamp	Resistive	600
2	Electric kettle	Resistive	1800
3	Air compressor	Inductive	950
4	Vacuum cleaner	Inductive	1200
5	Refrigerator	Inductive	250
6	Electric drill	Inductive	240
7	Electric hammer	Inductive	1200
8	Fluorescent lamp	Non-linear	11
9	Energy saving lamp	Non-linear	255
10	Microwave oven	Non-linear	1500
11	Computer screen	Non-linear	200
